# DrugQuest - a text mining workflow for drug association discovery

**DOI:** 10.1186/s12859-016-1041-6

**Published:** 2016-06-06

**Authors:** Nikolas Papanikolaou, Georgios A. Pavlopoulos, Theodosios Theodosiou, Ioannis S. Vizirianakis, Ioannis Iliopoulos

**Affiliations:** Division of Basic Sciences, University of Crete, Medical School, Gouves, 71003 Heraklion, Crete Greece; School of Pharmacy, Laboratory of Pharmacology, Aristotle University of Thessaloniki, University Campus, 54124 Thessaloniki, Greece

**Keywords:** Drug associations, Chemicals, Data integration, Name entity recognition, Text mining, Document clustering, Knowledge discovery

## Abstract

**Background:**

Text mining and data integration methods are gaining ground in the field of health sciences due to the exponential growth of bio-medical literature and information stored in biological databases. While such methods mostly try to extract bioentity associations from PubMed, very few of them are dedicated in mining other types of repositories such as chemical databases.

**Results:**

Herein, we apply a text mining approach on the DrugBank database in order to explore drug associations based on the DrugBank “Description”, “Indication”, “Pharmacodynamics” and “Mechanism of Action” text fields. We apply Name Entity Recognition (NER) techniques on these fields to identify chemicals, proteins, genes, pathways, diseases, and we utilize the TextQuest algorithm to find additional biologically significant words. Using a plethora of similarity and partitional clustering techniques, we group the DrugBank records based on their common terms and investigate possible scenarios why these records are clustered together. Different views such as clustered chemicals based on their textual information, tag clouds consisting of Significant Terms along with the terms that were used for clustering are delivered to the user through a user-friendly web interface.

**Conclusions:**

DrugQuest is a text mining tool for knowledge discovery: it is designed to cluster DrugBank records based on text attributes in order to find new associations between drugs. The service is freely available at http://bioinformatics.med.uoc.gr/drugquest.

## Background

The latest advances of next generation sequencing techniques, as well as the rise of the era of personalized medicine, have opened new challenges in the field of Bioinformatics. Data integration, drug discovery, drug repurposing, organization of chemical compound information in databases, identification of their therapeutic properties and their side effects along with the discovery of novel associations between them still remain active research fields.

There is a plethora of widely used databases that attempt to organize chemical information along with others which specialize in drug interactions. Herein, we present a short review of repositories which serve the former purpose. *PubChem* [1, 2], for example, is a database mainly composed by *PubChem Substance*, *PubChem Compound*, and *PubChem BioAssay* and is designed to provide information on the biological activities of small molecules. Today, PubChem hosts information for about 68,369,263 compounds, 196,730,517 substances, 1,154,333 BioAssays, 2,083,054 tested compounds, 3,141,545 tested Substances, 64 RNAi-BioAssays, 228,500,456 BioActivities, 9853 Protein Targets and 57,039 gene targets. *Chemical Entities of Biological Interest (ChEBI)* database [3, 4] is a freely available dictionary of molecular entities focused on small chemical compounds. *ChemExper* (www.chemexper.com) is a web based database which contains information about chemicals and their physical characteristics. *ChemExper* can be updated manually as everyone is allowed to submit new, update existing and retrieve chemical records online. *ChemBank* [5] is focused on incorporating small molecules, small-molecule screens and resources towards the gain of biological and medical insights. It is designed to aid chemists in synthesizing novel compounds and biologists in exploring small molecules that perturb specific biological pathways. *Side Effect Resource (SIDER)* [6] is a great collection of marketed medicines along with their recorded adverse drug reactions and their side effects. At the moment SIDER holds information about 996 drugs, 4192 side effects and 99,423 drug-side effect pairs. *ChemSpider* (http://www.chemspider.com) is a data integration platform which comes with a fast indexing/searching of over 26 million structures from hundreds of data sources. Its mission is to bring together information from 34 million compounds from over 490 data sources, along with their original source links. *Therapeutic Target Database (TTD)* [7] provides information about the known and explored therapeutic protein and nucleic acid targets, the targeted disease, pathway information and the corresponding drugs directed at each of these targets. This database currently contains 2025 targets (364 successful, 286 clinical trials and 1331 research targets) and 17,816 drugs (1540 approved, 1423 clinical trials, 14,853 experimental drugs and 3681 multi-target agents, 14,170 small molecules and 652 antisense drugs with available structure or oligonucleotide sequence). Targets and drugs in this database cover 61 protein biochemical classes and 140 drug therapeutic classes respectively. *SuperTarget/Matador* [8] is designed to give answers to complex queries such as finding drugs that are metabolized by the same enzyme, drugs that target a certain metabolic pathway or even drugs that target the same protein but are metabolized by different enzymes. The scenarios are based on information about medical indication areas, adverse side effects and drug metabolism. Currently, the database contains more than 2500 target proteins, which are annotated with about 7300 relations to 1500 drugs. Finally, *SuperDrug* [9] contains approximately 2500 chemical structures of active ingredients of essential marketed drugs. At the moment, it contains 2.396 compounds with 108.198 conformers.

In this article, we focus on the *DrugBank* [10–12] repository which is a freely available resource that combines detailed information about 7736 drug entries including 1584 FDA-approved small molecule drugs, 158 FDA-approved biotech (protein/peptide) drugs, 89 nutraceuticals and over 6000 experimental drugs. For each drug, information about taxonomy, pharmacology, pharmacoeconomics, chemical properties, related literature and other chemical interactors can be retrieved along with information about its targeted proteins.

DrugQuest clusters DrugBank records based on their textual information in a multidimensional vector space. We mainly apply partitional clustering algorithms in order to group together DrugBank records based on their textual information. Toxicity, targeted pathways, targeted proteins, diseases and/or other interactors are few examples of such textual information. Uniquely assigning DrugBank records into clusters, based on tagged terms such as pathways diseases, molecules, biological processes, can make *DrugQuest* a promising tool for new concept discovery and detection of new drug associations. The platform is available at http://bioinformatics.med.uoc.gr/drugquest.

## Methods

### An overview of DrugQuest’s workflow in steps

**Fig. 1:**
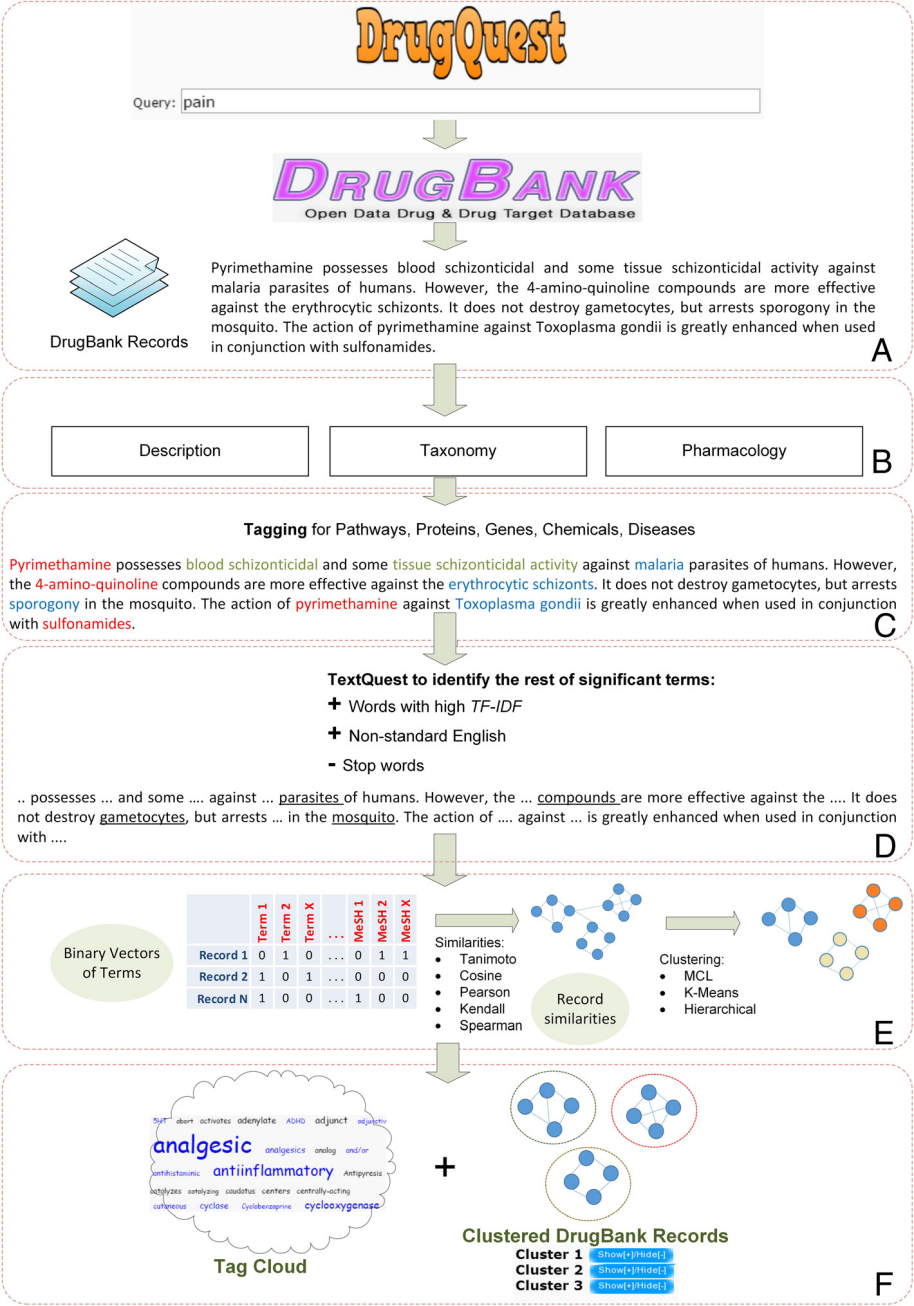
DrugQuest’s workflow. **a** Queries to DrugBank and retrieval of records related to the query. **b** DrugBank record mining based on textual information such as: description, toxicology and pharmacology. **c** Name Entity Recognition techniques to identify genes/proteins, chemicals, diseases, pathways. **d** TextQuest algorithm to identify non tagged Significant Terms. **e** Partitional clustering of DrugBank records using various clustering algorithms and similarity measures. **f** Visual representation of results: *Left*: Tag cloud example of highly representative terms per cluster. *Right*: DrugBank records assigned to clusters

The workflow of DrugQuest is summarized below in ten steps and presented analytically in Fig. 1.

1. The user provides a query (keyword matching using Boolean operators).
2. Selection of relevant DrugBank records upon query based on the “Description”, “Indication”, “Pharmacodynamics” and “Mechanism of Action” fields of the DrugBank records.
3. Retrieval of textual entries of the drug records from the local database, where DrugBank is stored.
4. Collection of tagged terms for each record. Notably, the tagging of the whole DrugBank repository has been performed beforehand, in order to avoid unnecessary bottlenecks for the user. DrugQuest uses the Reflect tagging service [13] to identify proteins and chemicals and the BeCAS tagging service for diseases/disorders and pathways identification.
5. Calculation of the TF-IDF score (Term Frequency x Inverse Document Frequency) for each of non-tagged words in the textual corpus to determine its ‘importance’.
6. Removal of English words with low TF-IDF values based on the British National Corpus (BNC - http://www.natcorp.ox.ac.uk/), a collection of samples of written and spoken language from a wide range of sources, accompanied by the respective word frequencies, designed to represent a wide cross-section of British English, both spoken and written, from the late twentieth century.
7. Removal of words belonging to a custom designed “stop word list” with common English words, such as articles and prepositions. The remaining words, after steps (4) - (7) will be characteristic for each abstract and will be referred as “S*ignificant Terms*”.
8. Creation of binary vectors representing each DrugBank record, indicating the presence or absence of Significant Terms and of tagged terms representing proteins, chemicals, diseases and pathways. In these vectors, the TF-IDF value is not taken into account.
9. Document clustering is performed with a user-defined combination of metric-clustering algorithm (selected among different available options).
10. Annotated representations and visualization of the results in two forms i.e. “*Tag Clouds*” and “*Clustered Drugs*” allow user to detect which of the terms belong to the four tagging categories.

### Query system

DrugQuest is a freely available, easy-to use web application which mines the DrugBank repository and clusters its records based on their textual information towards the discovery of new drug associations. It comes with a user friendly Google-like interface where one can query for a symptom (i.e. “pain”, “headache” etc.) and retrieve the relevant to the query DrugBank records. Notably, DrugQuest’s query system at the moment allows for simple keyword string matching within the textual information of each DrugBank record. Users can choose between simple Boolean operators (‘OR’ for any query term and ‘AND’ for all query terms). As each DrugBank record consists of various fields, we selected for fields with a high textual information content, more particularly: “Description”, “Indication”, “Pharmacodynamics” and “Mechanism of Action”.

### Automated identification of terms

Named Entity Recognition techniques have been applied on the locally stored and parsed DrugBank (version 4.2) repository. To minimize the gene/protein and chemical disambiguation problem and cope with the complexity of multiple synonyms, we link synonymous terms to unique database identifiers by utilizing the Reflect tagging service [13]. Similarly, for diseases and pathways we utilized the BeCAS tagging service [14]. This way, gene and protein names are mapped to ENSEMBL identifiers, drug/chemical names to PubChem [1, 2], diseases/disorders to a subset of UMLS [15] and pathways to the NCBI BioSystems repository [16]. Prior to using the tagged terms identified by both tagger, we manually checked for redundancies and inconsistencies.

In order to take advantage of the remaining untagged text, we utilize the TextQuest algorithm [17] to identify biologically significant words. Such words may refer to a phenomenon or a biological process or a function and might be worthy of attention. Shortly, the TextQuest algorithm initially calculates the TF-IDF score (Term Frequency x Inverse Document Frequency) for each word in the corpus to determine its ‘significance’. Then it removes the words with low TF-IDF scores and words belonging to a custom designed “stop word list” with common English words, such as articles and prepositions. The remaining words are characteristic for each abstract and we treat them as ‘Significant Terms’.

Ideally, a tagger should identify all synonyms and redirect them to the same database record. In the very rare cases where this does not occur, two synonyms may both appear as Significant Terms.

### Document clustering

Prior to partitional clustering, we represent each DrugBank record with a binary vector holding the presences and the absences of the tagged and the other/remaining biologically significant terms (not captured by the taggers) that were found in the text collection. Similarity metrics such as *Tanimoto coefficient*, *Pearson coefficient* or simple *cosine similarity* are then calculated in order to construct an all-against-all similarity matrix between the retrieved DrugBank records, relevant to a query. Based on this similarity matrix, we subsequently apply a partitional algorithm (among several algorithms that are available) to group the retrieved records and assign them to distinct clusters based on their textual information. At the moment, a plethora of clustering algorithms such as Affinity Propagation [18], MCL [19], *k*-Means [20], average linkage hierarchical clustering from SCPS [21] and spectral [22] clustering algorithms can be used.

### Representation of results and on-the-fly data integration

DrugQuest delivers different views of the results organized under tabs, along with a frame holding a summary of the analysis. The “*Tag Clouds*” view displays a tag-cloud of the Significant Terms that characterize each document cluster. The font size of each text is proportional to the frequency of the term in the respective cluster and, therefore, the bigger the size, the more over-represented the term. More specifically, the font size of each Significant Term is proportional to the number of records of each cluster in which the term appears. Terms that do not appear very often (based on an empirically chosen TF-IDF threshold of 19) in each cluster are not shown in order to present a less ‘cluttered’ and more user-friendly cluster. In this view, users can highlight terms that are unique for a cluster as well as tagged genes/proteins, chemicals, pathways, diseases and terms that are not standard English terms (i.e. they do not belong in the reference English dictionary). The “*Clustered Drugs*” tab categorizes the DrugBank records in subjects corresponding to implicit concepts accompanied with a link to the respective DrugBank record.

### Implementation and running time

DrugBank repository is stored locally in a MySQL database. The web interface is written with the use of CGI, Perl and Javascript. The MCL algorithm is written in C while the rest of the clustering algorithms in Java taken from the jClust java application [23]. Finally, vector similarities are calculated with the use of R package [24]. As DrugQuest has a limit of 5000 textual records per analysis, the running time complexity of the algorithms is not an issue. Moreover, due DrugBank’s small size, each query normally lasts few seconds to process.

## Results

### Pharmacological exploitation of DrugQuest usefulness through the example of the term ‘aspirin’

Aspirin (acetylsalicylic acid) is one of the most widely used drugs, since it has been in the market for more than 100 years (first synthesis and clinical trial in 1897–1899). Aspirin belongs to the pharmacological class of non-steroidal anti-inflammatory drugs (NSAIDs). The pharmacological mechanism of action of aspirin is mediated through the inhibition of both cyclooxygenases 1 and 2 (COX-1, COX-2), thus decreasing pain, fever, and inflammation. Interestingly, besides its well-known analgesic, antipyretic and anti-inflammatory activity, aspirin also exerts anticoagulant effects by inhibiting platelet aggregation. The favorable response of aspirin in reducing fever is mediated through the inhibition of prostaglandin E2 (PGE2) synthesis. A more recently developed class of NSAIDs is that of COX-2 specific inhibitors, such as celecoxib [25–28].

**Fig. 2:**
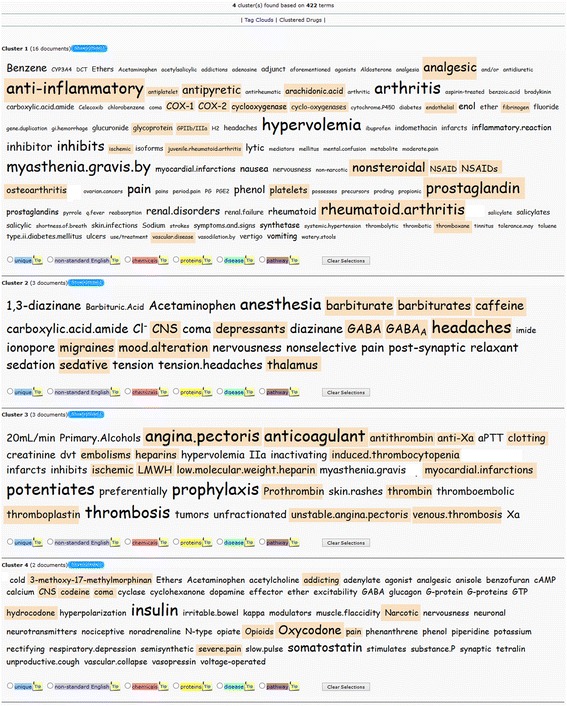
Aspirin Example. Tag Cloud view for term “aspirin” related query. *Cluster 1*: tags focusing on the anticoagulant blood effects of aspirin in related diseases including other anticoagulant drug classes along with analgesic, antipyretic and anti-inflammatory activities of aspirin. *Cluster 2*: tags refer to combination therapy of aspirin with other pharmacological classes of drugs. *Cluster 3*: tags propose combination therapy of aspirin with other analgesic drugs for the relief of pain in severe conditions. *Cluster 4*: tags point to a specific disease where aspirin is included in the therapeutic protocol, e.g. heart diseases

In the following example, by querying for “aspirin” in DrugQuest and using the MCL clustering algorithm with inflation value 3, the pharmacological usefulness of this text-mining biomedical suite is clearly displayed. The MCL algorithm calculates automatically the number of clusters, i.e. the user does not provide a preference bias for the number of clusters. As shown in Fig. 2, four clusters have been recovered. The analysis of tags grouped in each cluster revealed that: a) Cluster 1 consists of i) tags focusing on the anticoagulant blood effects of aspirin in related diseases including other anticoagulant drug classes (*tags: platelets, antiplatelet, thromboxane, glycoprotein, GPIIb/IIIa, IIb/IIIa, fibrinogen, endothelial, vascular disease, ischemic*) and ii) analgesic, antipyretic and anti-inflammatory activities of aspirin as well as the related diseases and other classes of relevant drugs (*tags: prostaglandin, anti-inflammatory, Cox-1, COX-2, non-steroidal, NSAIDs, NSAID, analgesic, antipyretic, rheumatoid arthritis, arachidonic acid, cyclooxygenase(s), cyclooxygenase, juvenile rheumatoid arthritis, osteoarthritis*); b) Cluster 2 tags refer to combination therapy of aspirin with other pharmacological classes of drugs (*tags: barbiturate(s), caffeine, CNS, GABA, GABA*_*A*_*, headaches, migraines, mood alteration, sedative, depressants, thalamus).* For example, as shown in DrugBank, “Butalbital is often combined with other medications, such as acetaminophen or aspirin, and is commonly prescribed for the treatment of pain and headache…Methylphenobarbital … and thiamylal are barbiturates …. often combined with aspirin”; c) Cluster 3 tags propose combination therapy of aspirin with other analgesic drugs for the relief of pain in severe conditions (*tags: oxycodone, coma, codeine, hydrocodone, addicting, opiods, narcotic, pain, severe pain, CNS, 3-methoxy-17-methylmorphinan*). For example, as shown in DrugBank, “Oxycodone… and hydrocodone are narcotic analgesics … often combined with aspirin”. d) Cluster 4 tags point to a specific disease where aspirin is included in the therapeutic protocol, e.g. heart diseases (*tags: angina pectoris, anticoagulant, antithrombin, anti-Xa, clotting, embolisms, heparins, induced thrombocytopenia, ischemic, LMWH, low molecular weight heparin, myocardial infractions, prothrombin, thrombin, thrombosis, thromboplastin, unstable angina pectoris, venous thrombosis*).

Overall, the classification of knowledge related to ‘aspirin’ by DrugQuest in these 4 clusters corresponds to the various levels of the existing pharmacological information for this old drug. Importantly, this information is appropriately categorized providing an overview of the drug in a way that could be useful for both research and educational purposes to healthcare practitioners, healthcare policy makers, regulatory agencies and pharmacologists.

### Detecting drugs belonging to the pharmacological class of selective serotonin-reuptake inhibitors (*SSRIs*)

Antidepressant drugs belonging to the class of selective serotonin-reuptake inhibitors (*SSRIs*) are used as an additional case study to further exemplify the usefulness of DrugQuest. In particular, very similar SSRI drugs such as *citalopram*, *fluoxetine*, *paroxetine* and *sertraline* were compared to each other in order to pinpoint differences and similarities. According to DrugBank, despite SSRIs act as potent inhibitors of neuronal serotonin re-uptake, they do not substantially affect norepinephrine or dopamine reuptake nor do they antagonize α- or β- adrenergic, dopamine D_2_ or histamine H_1_ receptors. In this manner, SSRIs affect somatodendritic 5-HT_1A_ and terminal autoreceptors that subsequently lead to adaptive changes in neuronal function, thus leading to enhanced serotonergic neurotransmission. Moreover, the clinical use of SSRIs can lead to the emergence of adverse drug reactions (ADRs), like dry mouth, nausea, dizziness, drowsiness, sexual dysfunction and headache [29–32].

**Fig. 3:**
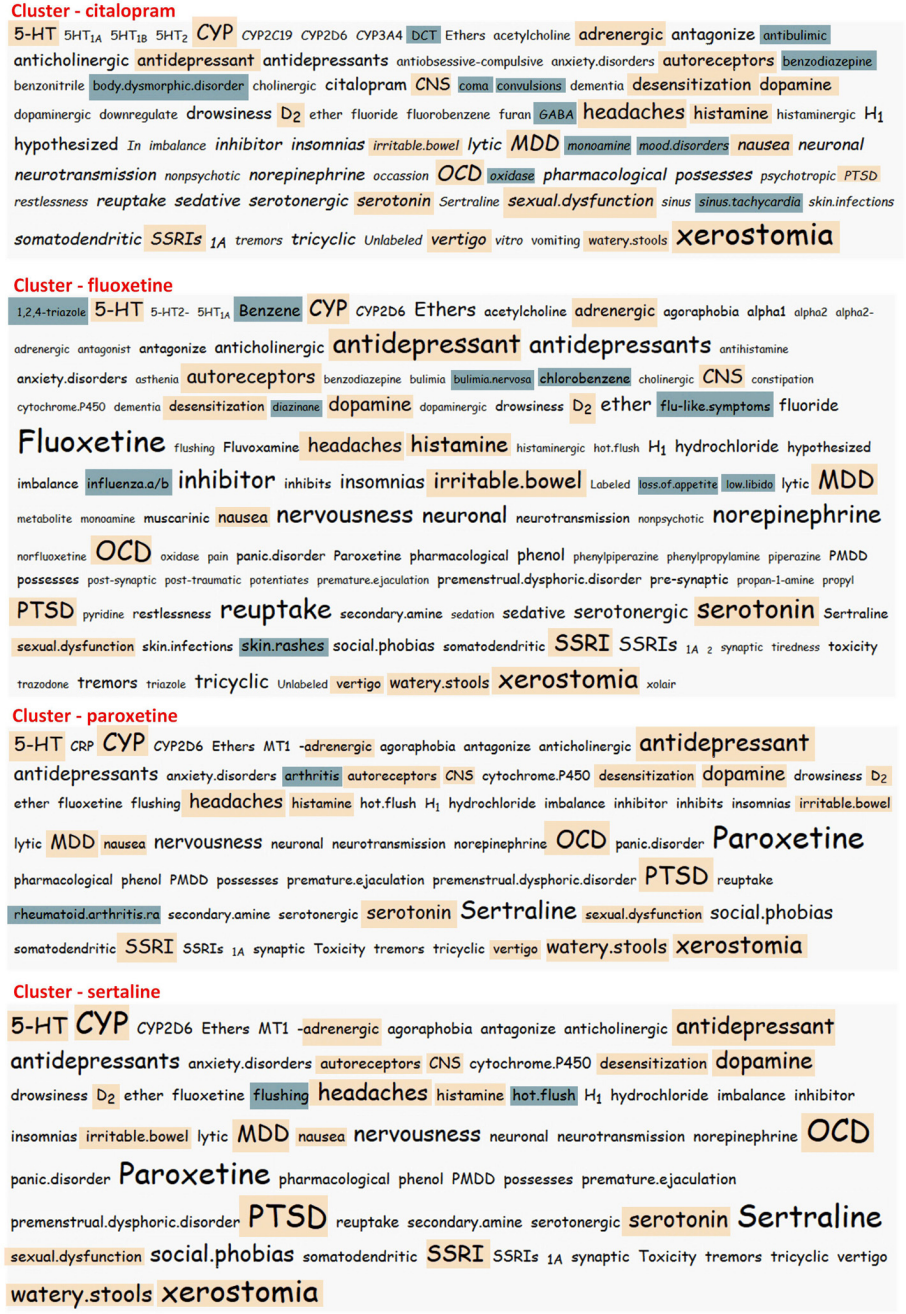
SSRIs Example. Tag Cloud view for drugs “citalopram*”*, “fluoxetine*”*, “paroxetine*”* and “sertraline*”. Orange*: common tags characterizing the class of SSRIs (in terms of pharmacological effects, ADRs, and/or clinical uses). *Blue*: tags related to specific pharmacological, chemical or clinical properties of each individual drug appearing in each respective cluster

As shown in Fig. 3, by querying for each of the aforementioned drugs and using the MCL algorithm, DrugQuest produced one cluster with Significant Terms for each of them. By inspecting the tag content of the relevant clusters we clearly observe two traits: i) common tags characterizing the class of SSRIs (in terms of pharmacological effects, ADRs, and/or clinical uses) appear in all four clusters (*5-HT, CYP, adrenergic, antidepressant, autoreceptors, CNS, desensitization, dopamine, drowsiness, D*_*2*_*, headaches, histamine, H*_*1*_*, irritable bowel, nausea, OCD, panic disorder, MDD, premature ejaculation, premenstrual dysphoric disorder, PTSD, reuptake, serotonergic, serotonin, sexual dysfunction, somatodendritic, SSRIs, tremors, vertigo, watery stools, xerostomia).* ii) tags related to specific pharmacological, chemical or clinical properties of each individual drug appearing in each respective cluster. The *Citalopram* cluster is uniquely characterized by the terms *DCT, antibulimic, benzodiazepine, dysmorphic disorder, coma, convulsions, GABA, monoamine, mood disorders, oxidase, sinus tachycardia*. Similarly, the *Fluoxetine* cluster is uniquely characterized by the terms *1,2,4-triazole, Benzene, bulimia nervosa, chlorobenzene, diazinane, flu-like symptoms, influenza, loss of appetite, low libido, skin rashes.* In the *Paroxetine* cluster, the terms *Arthritis, rheumatoid arthritis* are over-represented whereas in the *Sertraline* cluster, the terms *flushing, hot flush* are highlighted*.*

## Discussion

DrugQuest is a concept discovery tool mainly designed for finding new associations between known drugs but also for providing concise summation of a large corpus of drug-related knowledge. It uses textual information related to a drug and allows clustering algorithms to group chemicals based on this information. As it is chemically oriented, it differs significantly from its sister project BioTextQuest [33, 34], which is mainly developed to mine PubMed and cluster PubMed documents into topics. Among others, one of the main differences is that DrugQuest additionally uses tagging services at the back-end to cope with the complexity of multiple synonyms and chemical disambiguation, a feature that is missing in the BioTextQuest application.

To our experience and from a text mining point of view, chemical databases are peculiar in terms of the terminology and the vocabulary used, and small name changes may refer to completely different molecules with different properties and characteristics. Therefore, pre-defined tagging services were necessary to spot such details as opposed to BioTextQuest that mines textual corpuses more freely.

Despite the fact that the gene/protein annotation provided by the Reflect API and the Reflect Web Service [13] can vary, probably due to the backend dictionary updates, we insisted in using the API to pre-annotate the whole DrugBank. This allowed us to avoid invoking the external Reflect Web Service on the fly, sidestepping any Reflect Web Service downtime and time bottlenecks.

DrugQuest currently mines the DrugBank repository, but we aim to integrate other repositories like the ones mentioned in the introductory section (PubChem, ChEBI, SuperTarget/Matador etc.). Notably, we chose to start with the DrugBank database because of its smaller size, a fact that renders it easier for parsing. It is well maintained and many records are manually curated while it is frequently referenced by other resources.

Overall, we believe that the philosophy of DrugQuest could be a promising approach and a good starting point in mining chemical-related repositories and can boost the extraction of new knowledge by bringing unobserved drug repurposing and drug repositioning scenarios on the surface.

## Conclusions

DrugQuest is a web application that utilizes state-of-the-art text mining methodologies, name entity recognition techniques and data-integration approaches to mine the DrugBank repository and group chemicals/drugs based on their textual information.
